# Cyst of the Pancreas

**Published:** 1888-02

**Authors:** Chr. Fenger

**Affiliations:** Professor of Clinical Surgery, College of Physicians and Surgeons; 327 North Wells Street


### A CASE OF TRAUMATIC CYST OF THE PANCREAS.

BY CHR. FENGER, M. D.,

PROFESSOR OF CLINICAL SURGERY, COLLEGE OF PHYSICIANS AND SURGEONS.

[Reported, with statistics, by A. Holmboe, M. D.]

Chester A. King, æt. 8, was admitted to Emergency Hospital October 6, 1887, with the following history: He had enjoyed good health up to recent illness. Eleven weeks ago he fell from a horse and sustained injury to his abdomen, showing a red spot over the umbilicus, and a similar spot over the processus spinosus of third lumbar vertebra. He complained during first eight weeks of intermittent attacks of pain, located in the region about umbilicus. About three weeks after injury a swelling appeared in epigastrium and has been increasing steadily since. After injury the patient lost appetite; his bowels were loose, irregular, and the fæces of a whitish, curdled appearance, according to his mother's statement. Vomiting had been a prominent symptom during the period from the time of injury until his admission into the hospital. He had been treated for typho-malarial fever.

### STATUS PRÆSENS.

Patient is of normal size for his age, but thin and emaciated. Examination shows lungs and heart normal. In the epigastrium and upper half of mesogastrium is a round prominence caused by a tumor 7 inches in transverse diameter, 8 inches in longitudinal diameter. Percussion over the tumor is dull on the right side, continuous with the liver—dullness on the left side, reaching over the border of the ribs. Between the seat of dullness of the tumor and heart-dullness there is a tympanitic area from the ventricle. The circumference of abdomen at the end of xiphoid process, 24 inches; midway between this and the umbilicus, over the most prominent part of the tumor, 25 inches; at the umbilicus 23½ inches. After inflation of the bowel with Richardson's syringe the transverse colon is seen passing along and over the lower border of the tumor. The tumor moves with the respiration. There is transmitted pulsation from the aorta, but no expansion or bruit. Over the surface of the tumor is a feeling of fluctuation. The tumor is slightly movable from side to side.

Exploratory puncture brings out a syringeful of deep straw-colored, not perfectly clear, fluid of alkaline reaction, which contains a small number of red blood-corpuscles, but no other formed elements. The location of the tumor is in the median line behind the stomach, above the transverse colon—that is, in the region of the pancreas. Taken together with the microscopic character of its fluid contents, containing no other formed elements than a few blood-corpuscles, and finally its relation to a distinct trauma, made the diagnosis of a pancreatic cyst rather plausible. It was resolved to operate by laparotomy in two tempos, if the thickness of the cyst-wall would permit of such procedure. October 9, 1887, after the usual preparations for laparotomy, a longitudinal incision was made in the linea alba, about three inches long, midway between the umbilicus and the xiphoid process. After opening the peritoneal cavity the parietal peritoneum was stitched to the skin and the meso-colon transversum or ligamentum gastro-colicum was divided longitudinally and a few small vessels ligated. Beneath this was the glistening white smooth surface of the cyst-wall. This was united by a circular row of fine silk sutures, including a space about two inches long and one inch broad, to the abdominal wound. No cyst-fluid escaped along any of the sutures, and consequently the original plan of operating in two tempos was carried out. The wound-cavity was packed with iodoform-gauze, and antiseptic dressing was applied, held in position by an elastic bandage. The following day the temperature rose to 101.5° F., but came down to normal the evening of the same day and remained so. No other untoward symptoms followed this operation.

On October 16, a week after the laparotomy, the patient was again anæsthetized, and the cyst opened by Paquelin's cautery. The cyst-wall was of considerable thickness, almost one-fourth of an inch. About 40 ounces of thin, yellowish, semi-transparent fluid escaped. A large drainage-tube, over one-half inch in diameter and 9 inches long, was introduced to the bottom of the cyst, passing upwards and backwards toward the left side of the vertebral column, behind the stomach. No irrigation was used at the time of the operation, but heavy antiseptic dressing and an elastic bandage were applied.

The following day the temperature rose to 101° F., but became normal the next day and remained so. His bowels were regular before the second operation. After this, however, they did not move for five days, when an enema was given. Later on, the bowels again became regular. There was profuse discharge the first four to five days after the operation, after which time the discharge rapidly decreased in quantity. When leaving the hospital, November 9, the dressing which had been applied four days before was perfectly dry. At no time during the after-treatment was there any erosion or maceration of the skin surrounding the wound, although it was constantly in contact with the secretion from the cyst-wall.

The depth of the fistula at the time of leaving the hospital was three inches. The general condition of the patient was greatly improved.

In an article on Complicated Diseases of the Pancreas and their Surgical Treatment Dr. KARL HAGENBACH, assistant of Professor Socin, in Basel, has collected reports of fifteen cases of true cysts of the pancreas (*Deutsche Zeitschrift für Chirurgie*, December, 1887). Adding to these fifteen cases the reports of three cases operated upon in this country during latter part of 1887 (Bull, Fenger, Steele) we find, altogether, the following eighteen cases of cysts of the pancreas so far reported in literature:

| Literature. | Sex and Age. | Cause. | Duration. | Place of Development. | Symptoms. | Complications. | Diagnosis. | Method of operation. | Result. | Cyst-Contents. |
|---|---|---|---|---|---|---|---|---|---|---|
| 1. Gross. Anatomical Museum of Boston Society for Med. Improvement, 1847, p. 174. | M. 40 years.. | ........ | 13 years...... | Head of Pancreas. | Loss of strength, bloody stools, tumor in epigastr. | Icterus ....... | ........ | ........ | ........ | 300-400 Grm. serous bloody fluid. |
| 2. Salzer. Zeitschr. f. Heilk., 1886, Bd. vii, p. 11. | F. 42 years.. | ........ | 12 years...... | Tail of Pancreas. | Growing tumor. ........ | Icterus ....... | Ovarian cystoma... | ........ | ........ | 4,500 Grm. thick, slimy, grayish-brown fluid. |
| 3. Bosemann. N. Y. Med. Record, 1882, vol. xxi, p. 46. | F. 41 years.. | ........ | 7 years....... | Near the tail.. | Pain in regio-iliaca, emaciation, swelling of abdomen on left side. | ........ | Ovarian cystoma... | Extirpation of the pediculated cyst. | Healing in 38 days. | 10 liters brown fluid. |
| 4. Kulenkampff. Berl. klin. Wochenschr., 1882, 13 Febr., No. 7. | M. 39 years.. | Trauma..... | 6 months..... | ........ | Tenderness in epigastrium. | ........ | Echinococci of the liver. | Incision (2 tempos), drainage. | Healing in 6 weeks. | 1½-2 liters clear fluid. |
| 5. Dixon. N. Y. Med. Record, 1884, vol. xxv, p. 304. | M. 42 years.. | ........ | 11 weeks..... | Head of Pancreas. | Sudden pain in epigastrium, vomiting. | Icterus ....... | ........ | Puncture and aspiration. | Death 5 weeks after operation. | Abt. 300 Grm. light-yellow, slimy fluid, |
| 6. Senn. Journal of the American Med. Association, Sept. 26, and Oct. 3, 1885. | M. 19 years.. | Trauma...... | 5 weeks...... | Tail of Pancreas. | Pain, vomiting, emaciation, tumor. | ........ | Cyst of the pancreas. | Incision (1 tempo), drainage. | Healing in 7 weeks. | 3-3½ liters slightly opalescent fluid. |
| 7. Salzer. Zeitschr. f. Heilk., 1886, Bd. vii, p. 11. | F. 33 years.. | ........ | 11 years...... | Tail and body of pancreas. | Backache, vomiting, tumor in epigastrium. | ........ | Ovarian cystoma... | Extirpation of cyst. | Death 6 days after operation. | 5 liters yellowish-brown, clear fluid. |
| 8. Kramer. Centralbl. f. Chir., 1886, p. 23. | F. 16 years.. | ........ | Several weeks | ........ | Abdominal pain, tumor in epigastrium. | ........ | Echinococci of the liver. | Incision (1 tempo), drainage. | Healing in 4 months. | 2 liters clear fluid. |
| 9. Koetz. Guration ein Pankreascyste, Inaug. Diss. Marburg, 1886. | F. 36 years.. | ........ | 8 years....... | Tail......... | Enlargement of abdomen... | ........ | Ovarian cystoma... | Partial excision. | Death in 7 weeks. | 2½ liters brown, thick fluid. |
| 10. Kuester. Deutsche medic. Wochenshr., 1887, No. 10, p. 189. | M. 46 years.. | ........ | 6 months..... | ........ | Periodical gastric pains, loss of weight, tumor under right costal arch. | ........ | Cyst of the pancreas | Incision, tamponing, drainage. | Healing in 6 weeks. | 2½ liters clear yellowish fluid. |
| 11. Subotik. Allgem. Wiener medic. Zeitung, 1887, Bd. 32, p. 279. | M. 20 years.. | ........ | 3 years....... | ........ | Colic-like pains, vomiting, tumor in epigastrium. | ........ | Cyst of the pancreas | Incision (2 tempos), drainage. | Healing........ | 2 liters turbid brownish fluid. |
| 12. Riedel. v. Langenbeck's Archiv. f. klin. Chir., 1885, Bd. xxxii, p. 994. | F. 45 years.. | ........ | 9 years....... | Tail and body of pancreas. | Tumor in epigastrium...... | ........ | Ovarian cystoma... | Extirpation of the cyst. | Death 96 hours after operation. | 10 liters brownish fluid. |
| 13. Zukowsky. Wiener medic. Presse, 1881, No. 45. | F. 36 years.. | ........ | 2½ years..... | Tail.......... | Tumor in epigastrium...... | ........ | Ovarian cystoma... | Partial excision. | Death 10th day. | 5 liters brownish fluid, albumen, cholesterine. |
| 14. Thiersch. Berl. klin. Wochenschr., 1881, Bd. xvii, No. 40, p. 591. | M. 38 years.. | ........ | 1 year........ | Tail.......... | Took suddenly sick, tumor in region of the stomach. | ........ | Abscess of abdom. wall | Incision (2 tempos). | ........ | 3 liters chocolate-colored fluid. |
| 15. Gussenbauer. v. Langenbeck's Archiv. f. klin. Chir., 1883, Bd. xxix, p. 355. | M. 40 years.. | ........ | 11 weeks..... | ........ | Took suddenly sick with vomiting and gastric pain; emaciation, epigastric tumor. | ........ | Cyst of pancreas... | Incision (1 tempo), drainage. | Healing in 12 weeks. | 1,900 grm. grayish-black fluid. |
| 16. Bull. N. Y. Medical Journal, 1887, Oct. 1, p. 376. | M. 45 years.. | ........ | 10 months.... | Tail (?)....... | Sudden pain, vomiting, dyspnœa, loss of appetite, emaciation, stearrhœa. | Icterus, diabetes. | Cyst of the pancreas | Incision (2 tempos), drainage. | Recovery. (Death later from diabetes.) | 118 oz. Dark brown fluid. |
| 17. Fenger.......... | M. 8 years... | Trauma...... | 11 weeks..... | Tail......... | Pain in epigastr., vomiting, loss of appetite, emaciation, tumor in epigastr. region. | ........ | Cyst of the pancreas | Incision (2 tempos), drainage. | Healing in 2 months. | 40 oz. Straw-colored fluid. |
| 18. * Steele.......... | M. ........ | ........ | ........ | Tail......... | Tumor in epigastrium. | ........ | Cyst of the pancreas | ........ | Recovery....... | ........ |

* This case is not yet reported, which accounts for my meagre information concerning it.

From the above tabulated cases the following résumé may be drawn:*

There were eleven males and seven females.

Age from eight years (Fenger) to forty-six years (Küster).

Trauma was given as the cause in three cases ( Kulenkampff, Senn, Fenger).

Duration from five weeks (Senn) to thirteen years (Gross). In Kramer's case the time has been indefinitely given as "some weeks."

Place of development is not stated in five cases; in two cases the head is given as the seat of origin; in eleven cases the tail.

As to symptoms, a certain suddenness of onset is noticeable in a number of the cases; otherwise, common gastric and intestinal disturbances seem to be most prominent. Location, relations, etc., of tumor has only in a limited number of cases been sufficiently characteristic to warrant a diagnosis of pancreatic cyst (Senn, Küster, Subotik, Gussenbauer, Bull, Fenger, Steele).

Icterus is mentioned as a complication in four cases.

Diabetes in one case (Bull).

Sixteen cases were operated, of which ten recovered and five died.

Methods of operating † are distributed with the respective results as follows:

Extirpation of the whole cyst: three cases with two deaths and one recovery.

Partial excision of cyst-wall, uniting the remainder with the abdominal wound: two cases with two deaths.

Puncture and aspiration: one case with one death.

* For further details, see original reports.

† Method of operating employed in Dr. Steele's case was incision and drainage; but if in one or two steps, I do not know. Recovery.

Incision and drainage in one tempo: four cases with four recoveries.

Incision and drainage in two tempos: five cases with* five recoveries.

From the above figures it seems reasonable to conclude, as Dr. Senn stated in his monograph of 1885, that the operation to be recommended for cysts of the pancreas should be incision and drainage, and it seems to be a matter of little or no importance whether this operation is performed in one or two tempos.

Careful chemical analysis of the cyst-content has been made in several cases, showing the presence of tyrosin, leucin, mucin, serum-albumen, sodium, and potassium salts etc. The cyst-fluid has also proved itself able to digest starch, emulsify fat, etc., and thereby established its pancreatic origin. In Gussenbauer's and Senn's cases a digestion eczema was noticed around the abdominal wound. The quantity of the cyst-fluid varies from a pint up to several quarts. Blood in varying quantity is frequently found in these cysts. Küster considers the presence of blood characteristic, even for pancreatic cysts, when found in a cyst of the upper abdominal region (Hagenbach). In Thiersch's and Gussenbauer's cases, blood was prevalent to such an extent that Hagenbach considers it doubtful whether these two cases can, properly, be classed under retention-cysts, believing it possibly more correct to place them among hæmatomas. Senn, however, thinks it safe to classify them as hæmorrhagic retention-cysts.

* Bull's case was operated July 19, discharged cured November 19, but died two week's later from diabetes.

327 North Wells Street.

